# HIV and Antiretroviral Therapy Influence Tumor Microenvironment Architecture, Subclonal Evolution, and Immune Evasion Strategies in Diffuse Large B-cell Lymphoma

**DOI:** 10.1158/2767-9764.CRC-26-0343

**Published:** 2026-10-22

**Authors:** Jenny Coelho, Sophia M. Roush, Samantha Beck, Albert Wielgus, Alexander M. Xu, Amon Chirwa, Marriam Mponda, Edwards Kasonkanji, Maurice Mulenga, Tamiwe Tomoka, Satish Gopal, Akil Merchant, Matthew S. Painschab, Yuri Fedoriw

**Affiliations:** 1 Department of Pathology and Laboratory Medicine, School of Medicine, University of North Carolina (UNC), Chapel Hill, North Carolina.; 2 UNC Lineberger Comprehensive Cancer Center, Chapel Hill, North Carolina.; 3 Fischell Department of Bioengineering, University of Maryland, College Park, Maryland.; 4 UNC Project Malawi, Lilongwe, Malawi.; 5 Malawi Ministry of Health, Lilongwe, Malawi.; 6 National Cancer Institute Center for Global Health, Rockville, Maryland.; 7 Division of Hematology and Oncology, Department of Medicine, Cedars-Sinai Medical Center, Los Angeles, California.; 8 Department of Medicine, Indiana University School of Medicine, Indianapolis, Indiana.

## Abstract

The tumor immune microenvironment (TIME) and tumor heterogeneity shape prognosis and therapeutic response in diffuse large B-cell lymphoma (DLBCL), but human immunodeficiency virus (HIV)–associated (HIV+) DLBCL remains poorly understood. To characterize the TIME and tumor-intrinsic features of DLBCL across a spectrum of HIV-associated immune dysregulation, we applied imaging mass cytometry (IMC), spatial transcriptomics (ST), and whole-exome sequencing to 61 pretreatment DLBCL biopsies, stratified by HIV status and antiretroviral therapy (ART) exposure. In IMC, a tumor–immune–hot neighborhood was associated with inferior survival in HIV+ DLBCL (HR = 3.16; *P* = 0.013). Using ST, HIV+/ART-experienced tumors demonstrated greater macrophage infiltration (*P* = 0.02) and enrichment for M2 macrophage markers (CD163, MRC1, and MARCO) and PDCD1:CD274 coexpression. Unsupervised clustering identified 17 shared subclonal niches, with PRC1/BMI1 dysregulation in HIV+ tumors and PRC2/EZH2 enrichment in HIV-negative (HIV−) tumors. Pseudotime positioned HIV+/ART-naïve, HIV+/ART-experienced, and HIV− tumors at progressively later transcriptional states. CD47 increased over pseudotime in HIV+/ART-experienced tumors but decreased in HIV− tumors, whereas CD58 loss occurred in HIV− tumors only. In an exploratory paired exome subset, high−mutational burden tumors transitioned from proliferative to immune-engaged states, whereas low-burden tumors accumulated extracellular matrix and myeloid signatures suggesting fibrotic microenvironments. The data define distinct immune architectures in DLBCL across a spectrum of HIV-associated immune dysregulation. HIV and ART exposure seem to shape the DLBCL TIME in a manner that may reflect the degree of immune reconstitution during lymphomagenesis. The immunosuppressive, checkpoint-enriched microenvironment of ART-experienced tumors supports further investigation of immune checkpoint blockade and macrophage-targeting strategies in a population historically excluded from such trials.

**Significance:**

This study demonstrates that the systemic host immunity in which DLBCL arises influences the tumor microenvironment, tumor evolution, and immune evasion strategies that develop, with HIV+ DLBCL exhibiting earlier developmental states and selection for immunosuppressive environments. The findings provide biological rationale for further investigation of checkpoint therapy, and macrophage inhibitors, in HIV-associated lymphomas, despite historic exclusion of this population from immunotherapeutic clinical trials.

## Introduction

Diffuse large B-cell lymphoma (DLBCL) is the most common non–Hodgkin lymphoma (NHL) worldwide. Despite advances in chemoimmunotherapy, 30% to 40% of patients are refractory or relapse after first-line chemotherapy (1–3). This burden is especially pronounced in human immunodeficiency virus (HIV)–endemic regions, in which people with HIV (PWH) face up to 17-fold elevated DLBCL risk in the current era of antiretroviral therapy (ART), and lymphoma remains a leading cause of cancer-related mortality (4). Although histologically indistinguishable from HIV-negative DLBCL (HIV− DLBCL), HIV-associated DLBCL (HIV+ DLBCL) has distinct molecular features, including differences in mutational landscape and immune microenvironment composition (4–8). Yet, PWH have been underrepresented in research studies and clinical trials of novel therapeutics, creating a critical gap in our understanding and ability to apply cutting-edge treatment to this population (9–11).

The tumor immune microenvironment (TIME) has emerged as a key determinant of prognosis and therapeutic response in cancers, including DLBCL, independent of genomic profile (12–14). Immune checkpoint inhibitors targeting the *PDCD1*/*CD274* axis have shown limited efficacy in unselected relapsed/refractory DLBCL, and the composition and spatial organization of tumor-infiltrating immune cells are being increasingly recognized as predictive biomarkers for identifying target populations for therapeutic application (15–17). In HIV− DLBCL, tumors characterized by high immune infiltration have been associated with distinct outcomes that vary according to specific immune cell subsets and their spatial organization relative to malignant B cells (18).

Alongside the TIME, tumor-intrinsic features, including intratumoral heterogeneity and tumor mutational burden (TMB), shape both disease behavior and therapeutic response in DLBCL. Intratumoral heterogeneity is increasingly recognized as a driver of therapeutic resistance; approximately 84% of DLBCLs harbor detectable subclonal populations, and studies of paired tumors at diagnosis and relapse have identified distinct patterns of clonal evolution related to relapse timing (19, 20). Specific alterations in epigenetic regulators such as *KMT2D* and *CREBBP* represent early truncal events that may constrain subsequent tumor evolution, whereas mutations in immune evasion genes, including *CD58*, *B2M*, and *CD70*, are frequently enriched at relapse, suggesting that subclonal architecture and evolution are shaped by immune-mediated survival pressures (21–23). TMB, in contrast, has emerged as a biomarker of immunotherapy response, and HIV-associated malignancies harbor elevated TMB relative to HIV– counterparts (24–26). The processes contributing to subclonal diversity may also contribute to overall TMB, as genomic instability increases the rate of mutation accumulation and influences subclonal diversification (27–29).

The advent of spatial transcriptomics (ST) has further enabled high-resolution characterization of tumor−immune interactions by preserving spatial context while providing comprehensive molecular profiling (30–32). In DLBCL, spatial approaches have identified distinct microenvironmental niches associated with disease biology and clinical outcomes (33–35). Although extensive work has defined the prognostic relevance of the TIME, intratumoral heterogeneity, and TMB in HIV− DLBCL, these features remain largely uncharacterized in HIV+ DLBCL (9–11). Prior studies have demonstrated that HIV infection and ART alter the cellular composition of the DLBCL TIME, including decreased CD4^+^ T-cell infiltration and subsequently increased markers of exhaustion and CD8^+^ T-cell presence (6), but whether the prognostic relationships established in HIV− DLBCL are retained in the context of HIV-associated immune dysregulation remains unknown, as does how the immunologic environment of HIV infection influences subclonal architecture, clonal evolution, and TMB. Additionally, to our knowledge, ST profiling of HIV+ DLBCL to investigate the impact of HIV and ART on the TIME has yet to be performed.

In this study, we integrate ST, imaging mass cytometry (IMC), and whole-exome sequencing to systematically characterize both the spatial immune architecture and tumor-intrinsic features of DLBCL arising across varying degrees of HIV-associated immune dysregulation. Leveraging a longstanding cohort of patients with DLBCL in Malawi, a country with a high HIV burden and longstanding ART programs, we stratify tumors by HIV status and ART exposure to capture distinct immunologic contexts. We first map the spatial organization of the TIME and identify microenvironmental phenotypes with therapeutic implications and then investigate tumor-intrinsic subclonal architecture, evolutionary trajectories, and immune evasion strategies known to be shaped by each immunologic setting.

## Materials and Methods

### Clinical cohort

Tumor samples were collected as part of the Kamuzu Central Hospital Lymphoma Study, in Lilongwe, Malawi, an observational cohort of patients with newly diagnosed lymphoproliferative disorders (NCT02835911), as previously described (6, 36, 37). All patients met the following inclusion criteria: enrollment between 2013 and 2019, more than 18 years of age at enrollment, and histologically confirmed *de novo* DLBCL. All patients received standard-of-care CHOP (cyclophosphamide, doxorubicin, vincristine, and prednisone), and a subset (*n* = 19) received rituximab as part of a previously reported clinical trial (NCT02660710; ref. 38). Rituximab was not part of the standard of care for DLBCL in Malawi during the enrollment period and was therefore administered only to patients co-enrolled in the referenced clinical trial; its distribution across HIV/ART groups is shown in Table 1. At the time of lymphoma diagnosis, patients with HIV either continued or initiated ART. Patients were followed for up to 5 years with data censoring in March 2023.

**Table 1.** Cohort demographics.

​	HIV− (*N* = 23)	HIV+/ART-exp (*N* = 23)	HIV+/ART-naïve (*N* = 15)	Total (*N* = 61)
Age (years)	​	​	​	​
Median (Min, Max)	48 (25, 77)	46 (27, 57)	47 (22, 62)	47 (22, 77)
Sex	​	​	​	​
Female	7 (30.4%)	7 (30.4%)	8 (53.3%)	22 (36.1%)
Male	16 (69.6%)	16 (69.6%)	7 (46.7%)	39 (63.9%)
Prior ART (months)	​	​	​	​
Median (Min, Max)	NA	69.2 (10.9, 130)	0.986 (0, 4.57)	34.2 (0, 130)
CD4 count	​	​	​	​
Median (Min, Max)	NA	189 (68, 844)	199 (41, 392)	194 (41, 844)
HIV RNA	​	​	​	​
Median (Min, Max)	NA	0 (0, 24,600)	4,140 (0, 333,000)	0 (0, 333,000)
COO	​	​	​	​
GC	12 (52.2%)	12 (52.2%)	12 (80%)	36 (59%)
Non-GC	11 (47.8%)	11 (47.8%)	3 (20%)	25 (41%)
EBER	​	​	​	​
Positive	1 (5%)	0 (0%)	1 (6.7%)	2 (3.5%)
ECOG score	​	​	​	​
0–1	11 (47.8%)	13 (56.5%)	13 (86.7%)	37 (60.7%)
2–4	12 (52.2%)	10 (43.5%)	2 (13.3%)	24 (39.3%)
aaIPI score	​	​	​	​
0–1	9 (52.9%)	5 (23.8%)	7 (58.3%)	21 (42%)
2–3	8 (47.1%)	16 (76.2%)	5 (41.7%)	29 (58%)
IMC	20 (87%)	22 (95.7%)	13 (86.7%)	55 (90.2%)
ST	10 (43.5%)	12 (52.2%)	10 (66.7%)	32 (52.5%)
Treatment	​	​	​	​
CHOP	18 (78.3%)	14 (60.9%)	10 (66.7%)	42 (68.9%)
R-CHOP	5 (21.7%)	9 (39.1%)	5 (33.3%)	19 (31.1%)
Survival (months)	​	​	​	​
Median (Min, Max)	11.1 (0.0657, 60)	9.99 (0.263, 60)	52.8 (0.329, 60)	12.6 (0.0657, 60)

Abbreviations: aaIPI, age-adjusted IPI (available for *n* = 50 patients); ECOG, Eastern Cooperative Oncology Group.

Patients were stratified into three groups: HIV−, HIV+/ART-experienced (HIV+/ART-exp; prescribed ART at least 6 months prior to lymphoma diagnosis), and HIV+/ART-naïve (prescribed ART for less than 6 months at lymphoma diagnosis). Although there is significant variability in response to ART, the 6-month cutoff identifies clinically and biologically distinct groups, as previously demonstrated (6–8, 24).

Formalin-fixed, paraffin-embedded (FFPE) tissue blocks from diagnostic, pretreatment excisional biopsies were used for all molecular analyses. Epstein–Barr virus (EBV) positivity was determined using EBER-ISH as previously described; only two cases were EBER-positive (5, 6). Cell-of-origin (COO) classification was defined by IHC using the Hans algorithm as previously described (5, 39).

The study was approved by the University of North Carolina (UNC) Institutional Review Board and the Malawi National Health Sciences Research Committee, and all patients provided written informed consent.

### IMC

IMC data were available from a previously published study for 55 of 61 tumors (panel data in Supplementary File S1; ref. 6). Nearest-neighbor analysis (k = 20) identified six distinct cellular niches, labeled by the predominant population of each cell type: tumor–immune–hot, tumor-rich, stroma-rich, myeloid/CD8-rich, CD8-rich, and CD4-rich. The tumor–immune–hot neighborhood was defined by the greatest degree of mixing between immune and tumor cells and was predominantly localized to the tumor–stroma interface, consistent with a region of immune engagement with the tumor compartment. Tumors were subsequently classified as “immune-hot” or “immune-cold” using a median cutoff on the proportion of cells assigned to the tumor–immune–hot neighborhood. This classification reflects only the presence and relative abundance of colocalized immune and tumor cells, leading to the subsequent analyses utilizing ST.

PD-1/PD-L1 coexpression was assessed in the previously published IMC data. Per-cell PD-1 and PD-L1 intensities (normalized to 0–1) were correlated across all cells (Spearman). To approximate Visium resolution, cells within each region of interest were binned into contiguous 55-μm pseudo-spots (spots with ≥5 cells retained); spot-level coexpression was quantified as the Spearman correlation of spot-mean PD-1 and PD-L1 and as the fraction of pseudo-spots containing both a PD-1+ and a PD-L1+ cell (>0.5 normalized intensity, matching the phenotyping threshold). Cell-level colocalization was assessed by nearest-neighbor distance from PD-1+ CD8 T cells to PD-L1+ tumor and macrophage cells. HIV/ART comparisons used patient-level means with Kruskal–Wallis tests.

### ST

ST was performed on 32 tumors using the 10x Genomics Visium FFPE v2 with CytAssist on an 11 × 11–mm capture window (RRID: SCR_023571 and RRID: SCR_024570). Sample fixation and staining, probe hybridization, library preparation, and sequencing were performed according to manufacturer’s instructions by the Pathology Services Core, the Translational Genomic Laboratory (RRID: SCR_025231), and the Advanced Analytics Core facilities at UNC. Twenty-six of the 32 tumors had paired IMC data.

### Data preprocessing

All sequenced libraries were processed and aligned to the GRCh38 human reference genome using the 10x Genomics Space Ranger pipeline (RRID: SCR_025848). Data were imported to R and processed using the R package Seurat (ver. 5.1.0; RRID: SCR_016341; ref. 40). Trimming and filtering were performed (counts >10K/spot, features >200/spot, and mitochondrial genes <30%). Each sample was normalized using SCTransform, and datasets were merged using FindIntegrationFeatures, with batch correction performed using Harmony (RRID: SCR_022206; refs. 41, 42). Two tumors (T04 and T09) were identified as outliers because of very few spots per sample and lower median genes per spot and were included in pooled analyses but consequently excluded from between–tumor gene expression analyses (Supplementary Fig. S1). Additionally, T32, an EBV-positive tumor with histiocyte-rich features, was excluded from STanalyses because of the overwhelming myeloid signature which prevented identification of other cell types.

The spot size of the 10x Visium platform is 55 μm, with a 100-μm center-to-center distance. Considering the histologic architecture and cytologic features of DLBCL, manual supervised spot-labeling was performed to identify and assign predominant cell types per spot. Using Seurat’s AddModuleScore function, a signature score was calculated for each cell type in each spot using a simplified panel of established gene expression markers (Supplementary Table S1). Each spot was subsequently labeled by the highest-scoring cell type. Because DLBCL is a diffuse proliferation of malignant B cells and Visium spots lack single-cell resolution, malignant B cells could not be reliably distinguished from nonmalignant B cells; all B cell–dominant spots were therefore analyzed together as tumor-dominant spots, and each cell-type label reflects the predominant population within a spot rather than a pure cell population.

For COO analysis, COO was additionally determined by RNA sequencing using ST data in a spot-specific approach, using the gneseqCOO package in R (43).

### Whole-exome sequencing

Exome data were available from a previously published study for a subset of six tumors (24). DNA was extracted from pretreatment FFPE tumors (GeneRead DNA FFPE Kit) and corresponding whole-blood samples (QIAmp Blood Mini Kit). Target capture was performed using the Agilent SureSelect Human All Exon V6 r2 and libraries were sequenced on the NovaSeq 6000 platform. UNC Lineberger Bioinformatics Core’s jUNCtion pipeline was used for processing. Variants passing at least one variant caller’s filters were included and further filtered (tumor and germline read depth ≥15, germline alternative count <1, and tumor alternative count ≥5). Silent, intronic, and common variants were excluded, as were variants with variant allele frequency ≤5%, to account for FFPE artifacts (43). Tumors were classified as TMB-high or TMB-low by a median cutoff (median = 94 mutations, Supplementary Table S2).

### Spatial classification of the TIME

Using the spot-labeling described above, spots were categorized as immune (CD8^+^ T cells, CD4^+^ T cells, T follicular helper cells, and macrophages), tumor (B cells), or stromal (endothelial cells). Four spatial metrics were calculated using a 50-μm neighborhood radius, identifying adjacent spots. (i) Infiltration score was defined as the proportion of immune spots for which nearest tumor spot fell within the radius, computed via nearest-neighbor search (RANN package). (ii) Mixing score was calculated by identifying all neighbors within the radius for each tumor and immune spot (k ≤ 20) and computing, per spot, the fraction of opposite-type neighbors (tumor neighbors for immune spots and immune neighbors for tumor spots); these per-spot fractions were then averaged across all tumor and immune spots in the sample. (iii) Immune dispersion was quantified using the Ripley K function (spatstat package, *Kest* with border correction) evaluated over distances from 0 to twice the radius; the mean deviation of the observed K from the theoretical K under complete spatial randomness was computed and sign-inverted such that higher values reflect greater dispersion (and lower values reflect clustering) of immune spots. (iv) Interface thickness was defined as the proportion of all tumor and immune spots having at least one opposite-type spot within the radius, capturing the breadth of the tumor–immune contact zone. Each of the four metrics was *z*-score normalized across samples and averaged to produce a composite spatial hot score, with samples scoring above zero classified as spatially hot and those at or below zero as spatially cold. Tumors with no detected immune spots were assigned a score of zero for all metrics and classified as spatially cold.

### Peripheral and intratumoral CD4 analysis

To assess whether CD4 T-cell status accounted for differences in immune architecture across HIV/ART groups, peripheral and intratumoral CD4 measures were compared across groups and across spatial immune classifications. Peripheral CD4 count (cells/μL) and HIV RNA at diagnosis were obtained from the clinical record. Intratumoral CD4 content was quantified from IMC as the proportion of CD4^+^ T cells among all cells and from Visium as the proportion of CD4^+^ T cell–labeled spots. The peripheral CD4 count was correlated with intratumoral CD4 content, CD4-rich neighborhood proportion, and tumor–immune–hot neighborhood proportion by Spearman correlation with Benjamini–Hochberg correction. Intratumoral CD4 content (IMC and Visium) was compared across HIV/ART groups by Kruskal–Wallis tests with pairwise Wilcoxon *post hoc* tests, and peripheral and intratumoral CD4 were compared between spatially immune-hot and immune-cold tumors by Wilcoxon rank-sum tests (Supplementary Fig. S2).

### Gene expression analyses

#### Cell-type heatmap

Cell-type markers were identified using Seurat’s FindAllMarkers [positive enrichment only, ≥25% expression, and log_2_ fold change (FC) ≥ 0.25]. The top 10 markers per cell type by average log_2_FC were selected, and average expression per cell-type sample was log-transformed, *z*-score normalized by row, and visualized using pheatmap with hierarchical row clustering.

#### Principal component analysis and differentially expressed genes of HIV+ tumor clusters

Tumor-level expression profiles were generated from SCTransform-normalized data. Principal component analysis (PCA) was performed, optimal cluster number was determined by silhouette analysis, and k-means clustering was applied to principal component 1 (PC1) to PC2 coordinates. Differential expression used a pseudobulk approach with DESeq2 (*P* adjusted < 0.05).

#### Immune-hot versus -cold tumor differential gene expression

Two complementary approaches were used. Within-tumor differentially expressed genes (DEG) between hot and cold spots were identified per tumor using Wilcoxon rank-sum tests (FindMarkers; min.pct = 0.1 and log_2_FC ≥ 0.25). Meta-analysis identified genes significant (adjusted *P* < 0.05) in ≥3 tumors with consistent directionality. Between-tumor significance thresholds were Benjamini–Hochberg–adjusted *P* < 0.05 and |log_2_FC| > 0.5.

#### Differential gene expression of interface spots by HIV/ART status

Interface spot DEGs were identified using FindAllMarkers with additional filtering for robustness (≥10% expression within samples and present in ≥25% of tumors per HIV/ART group) and consistency (present in ≥30% of all tumors and coefficient of variation < 2).

#### Pseudobulk differential gene expression of tumor spots

Tumor-dominant spots were filtered to retain those with ≥200 total counts. Gene filtering was applied in three stages: (i) genes were retained if they had ≥100 counts in at least 25% of samples or total counts exceeding 50 across all samples, (ii) genes were required to be expressed in at least 20% of samples within at least one comparison group, and (iii) genes for which a single sample contributed more than 80% of total expression were removed. Differential expression was performed using DESeq2 (v1.38.0), with significance at Benjamini–Hochberg–adjusted *P* < 0.05 and |log_2_FC| > 0.28.

### Subclonal niche identification

Tumor-dominant spots were subset from the merged Seurat object and independently renormalized, and the top 2,000 variable features were selected via VST. Following scaling and PCA, Harmony batch correction was applied on sample identity. Uniform Manifold Approximation and Projection (UMAP) and graph-based clustering (Louvain, resolution 0.5) were performed to identify shared subclonal niches (SN). Low-abundance clusters present in fewer than 10 tumors were removed. DEGs between retained clusters were identified using FindAllMarkers (minimum percent expressed = 25% and log_2_FC threshold = 0.25). As Visium does not capture single-cell resolution, clusters were considered SNs representing regions comprising tumor subclones and their potential associated immune infiltrate. We use the term “subclonal niche” to denote transcriptionally distinct spatial regions; these designations are based on transcriptional state and do not, on their own, establish genetically defined subclones.

Individual tumor analyses were performed independently using the same clustering approach. For the TMB comparison, TMB-high and TMB-low tumors were analyzed as separate pooled groups with SCT integration and Harmony batch correction.

### Pathway and gene set enrichment analyses

Gene set enrichment analysis (GSEA) was performed on full ranked gene lists from DESeq2 results against the Molecular Signatures Database (MSigDB) C6 oncogenic signatures collection using clusterProfiler (RRID: SCR_016884) with Benjamini–Hochberg correction (44). For the pooled analysis, enrichment was additionally performed per cluster using the enricher function with all detected genes as the background universe. Gene symbols were converted to Entrez IDs via biomaRt (RRID: SCR_019214).

### Pseudotime analysis

Pseudotime trajectories were constructed for tumor-dominant spots and SNs using Monocle3 (RRID: SCR_016339). Seurat objects were converted to cell_data_set objects using SCT-normalized counts, and principal graph trajectories were learned independently for each tumor and merged datasets. Spearman rank correlation was computed between each gene’s expression and pseudotime, with Benjamini–Hochberg correction (q < 0.05). Pseudotime was normalized to a 0 to 1 scale within each sample and divided into 50 equally spaced bins. Group-level mean expression over pseudotime was visualized using LOESS-smoothed curves (span = 0.3). Pseudotime and trajectory analyses are based on transcriptional similarity and represent an inferred transcriptional ordering of tumor states rather than genetically defined clonal evolution or true temporal progression.

### Statistical analyses

All analyses were performed in R (v4.5.0). Two-group comparisons used Wilcoxon rank-sum tests; three-group comparisons used Kruskal–Wallis tests with pairwise Wilcoxon *post hoc* tests. Overall survival (OS) was analyzed using Kaplan–Meier curves with log-rank tests and Cox proportional hazards regression for hazard ratios [95% confidence interval (CI)]. Multivariable Cox models were additionally adjusted for ART status, rituximab treatment, age, COO, and age-adjusted International Prognostic Index (IPI) scores. For differential expression, Benjamini–Hochberg–adjusted *P* < 0.05 defined significance.

## Results

### Cohort characteristics

The full cohort consisted of 61 patients with DLBCL from Malawi (*n* = 23 HIV−, *n* = 23 HIV+/ART-exp, and *n* = 15 HIV+/ART-naïve; Table 1). ART-exp patients received ART for a median of 69.2 months prior to diagnosis compared with a median of 0.99 months in ART-naïve patients. Peripheral CD4 counts at diagnosis were comparable between the two HIV+ groups, whereas HIV RNA was significantly higher in ART-naïve than ART-exp patients (Supplementary Fig. S3). EBV positivity was identified in two tumors (3.3%). COO by Hans classifier was 59% germinal center (GC) and 41% non-GC, with no significant difference by HIV/ART status, although HIV+/ART-naïve tumors tended toward GC classification (80%). All patients received CHOP; 19 also received rituximab. HIV+/ART-naïve patients had improved 5-year OS compared with HIV− and HIV+/ART-exp groups (median 52.8 months vs. 11.1 and 9.99 months, respectively), as previously reported (6).

Of the 61 tumors, IMC was performed on 55 and ST profiling was performed on 32 (*n* = 10 HIV+/ART-naïve, *n* = 12 HIV+/ART-exp, and *n* = 10 HIV−). Twenty-six had paired IMC and ST profiling (Table 1). Six tumors (*n* = 2 HIV+/ART-naïve, *n* = 3 HIV+/ART-exp, and *n* = 1 HIV−) had paired exome sequencing data.

### IMC identifies tumor–immune neighborhoods associated with outcomes

**Figure 1.**
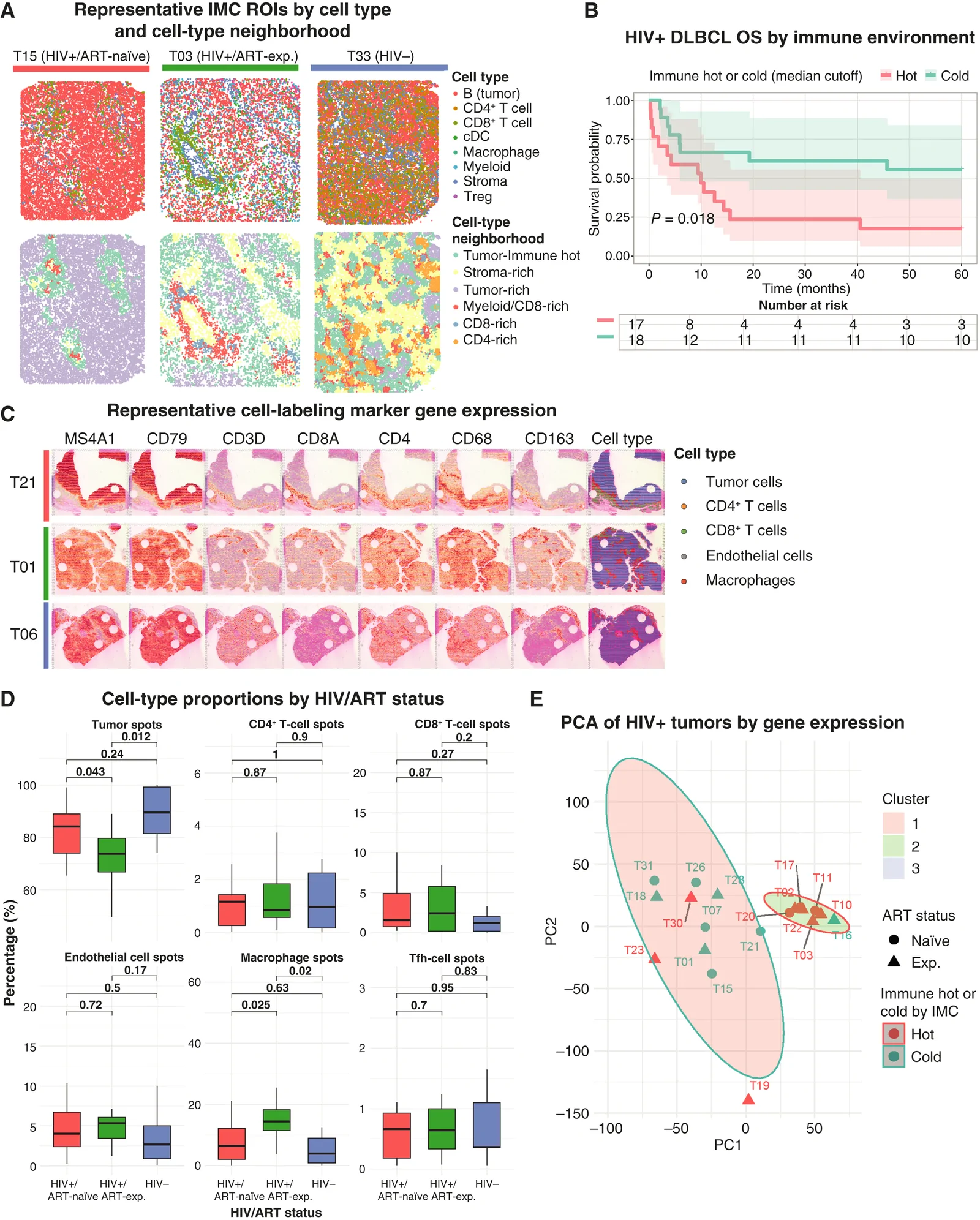
Spatial immune characterization of DLBCL by IMC and ST. **A,** Representative regions of interest of IMC data of three representative tumors. HIV+/ART-naïve (T15, red), HIV+/ART-exp (T03, green), and HIV− (T33, blue). In the top three panels, each spot represents a single cell and is colored by cell type. In the bottom three panels, each spot represents a single cell and is colored by cell-type neighborhood (k = 20 and center = 6). **B,** Kaplan–Meier survival curve of tumor–immune–hot neighborhood presence by median cutoff [above median = “Hot” (red); below median = “Cold” (blue)] in HIV+ DLBCL (log-rank *P* = 0.018; Cox HR = 3.16; *P* = 0.013). **C,** Gene expression of select markers used for spot-labeling of three representative tumors. HIV+/ART-naïve (T21, red), HIV+/ART-exp (T01, green), and HIV− (T06, blue). Spots colored by dominant cell-type assignment in the final panel. **D,** Comparison of cell types present in tumor by spot-labeling, normalized to total number of spots per tumor, by HIV/ART status (*n* = 31, Wilcoxon rank-sum test). **E,** PCA plot of HIV+ tumors by ST identified three clusters. Each tumor is colored by tumor–immune–hot neighborhood presence assigned by IMC using median cutoff, as in **B** [above median = “high” (red); below median = “low” (blue)], and shaped by ART status (circle = ART-naïve; triangle = ART-exp.). Clusters are denoted by colored ellipses (cluster one = red, cluster 2 = green, and cluster 3 is one tumor, T19). cDC, conventional Dendritic Cells.

To first establish the spatial immune architecture of DLBCL across the cohort, we leveraged previously published IMC data (*n* = 55 tumors) to identify cell-type neighborhoods. Six shared neighborhoods were identified across the cohort and defined by the dominant cellular population: stroma-rich, tumor-rich, myeloid/CD8-rich, CD8-rich, CD4-rich, and tumor–immune hot, which comprised mixed tumor and immune cell populations and localized to the tumor–stroma interface (Fig. 1A; Supplementary Fig. S4A). HIV+ tumors had significantly fewer CD4-rich neighborhoods than HIV− tumors (*P* < 0.001), with no difference by ART status (*P* = 0.39; Supplementary Fig. S4B); no other neighborhoods differed significantly by HIV or ART status. The tumor–immune–hot neighborhood was the only niche significantly associated with outcomes: tumors classified as immune-hot (above the median proportion of cells assigned to this neighborhood) demonstrated inferior survival in HIV+ DLBCL, independent of ART status (HR = 3.16; *P* = 0.013; Fig. 1B). In a multivariable Cox model adjusting for rituximab treatment, the immune-hot association with inferior survival was retained (adjusted HR = 2.81; 95% CI, 1.17–6.78; *P* = 0.021; Supplementary Table S3). This association remained significant after individual adjustment for ART exposure, age, COO, and age-adjusted IPI (adjusted HR, 2.69–5.19; all *P* < 0.05; Supplementary Table S3), and modeling the tumor–immune–hot neighborhood as a continuous variable (per doubling of hot-neighborhood abundance) similarly demonstrated progressively increasing risk that was robust to the same adjustments (HR, 1.44–2.05; all *P* < 0.05; Supplementary Table S3).

### ST resolves immune architecture and demonstrates increased macrophage infiltration in ART-experienced tumors

Because the IMC-defined tumor–immune–hot neighborhood was classified by spatial colocalization of tumor and immune cells rather than their functional state, the cell populations and transcriptional programs underlying its negative prognostic association in HIV+ DLBCL remained undefined. To resolve these features, we performed ST on 32 tumors, 26 with paired IMC. Supervised spot-labeling of ST data identified distinct cell-type populations affirmed by differential expression of genes outside the labeling panel (Supplementary Fig. S5). Tumor spots expressed canonical B-cell markers (*CD22* and *CD79B*) and GC-DLBCL–associated genes (*AFF2* and *DTX1*). Macrophage spots expressed monocyte/macrophage markers (*ADGRE4*, *FCGR3A*, and *VSIG4*), M2 marker *CD163*, and tumor-associated macrophage (TAM) gene *SPP1*. T-cell spots shared a T-cell receptor component (*TRBC1*), cytotoxicity markers (*GZMK* and *GZMA*), and T-cell developmental and regulatory markers (*GIMAP7* and *THEMIS*). Spot-labeling revealed immune architecture typically unidentifiable by hematoxylin and eosin staining because of the diffuse histologic nature of DLBCL (Fig. 1C).

Normalized spot proportions revealed fewer tumor spots in HIV+/ART-exp tumors compared with those in HIV− (*P* = 0.04) and HIV+/ART-naïve (*P* = 0.01), with specific enrichment for macrophage spots (*P* = 0.03 and *P* = 0.02, respectively) and overall TIME proportion (combined presence of CD4^+^ T cells, CD8^+^ T cells, and macrophages; *P* = 0.04 and *P* = 0.02; Fig. 1D; Supplementary Fig. S6). No differences were observed for other cell types.

### IMC-derived immune classification distinguishes ST clusters in HIV+ tumors

Having established the cellular composition of the TIME by ST, we next asked whether the IMC-defined immune classification associated with inferior survival in HIV+ DLBCL is reflected in tumor-level transcriptomic structure. PCA restricted to the 19 HIV+ tumors with paired IMC data revealed three distinct clusters that largely aligned with IMC-derived immune-hot versus immune-cold labeling (Fig. 1E). However, DEGs between clusters were limited. Cluster 1 (primarily cold) expressed cancer testis antigens (*MAGEB18* and *XAGE3*), *NOTCH1*, and *ZDHHC11B*; cluster 2 (primarily hot) expressed a different set of cancer testis antigens (*MAGEA1* and *SPANXN4*); and cluster 3 comprised a single tumor with no clear enrichment (Supplementary Fig. S7).

### ST independently defines immune-hot tumors and identifies myeloid and immunosuppressive transcriptional signatures

**Figure 2.**
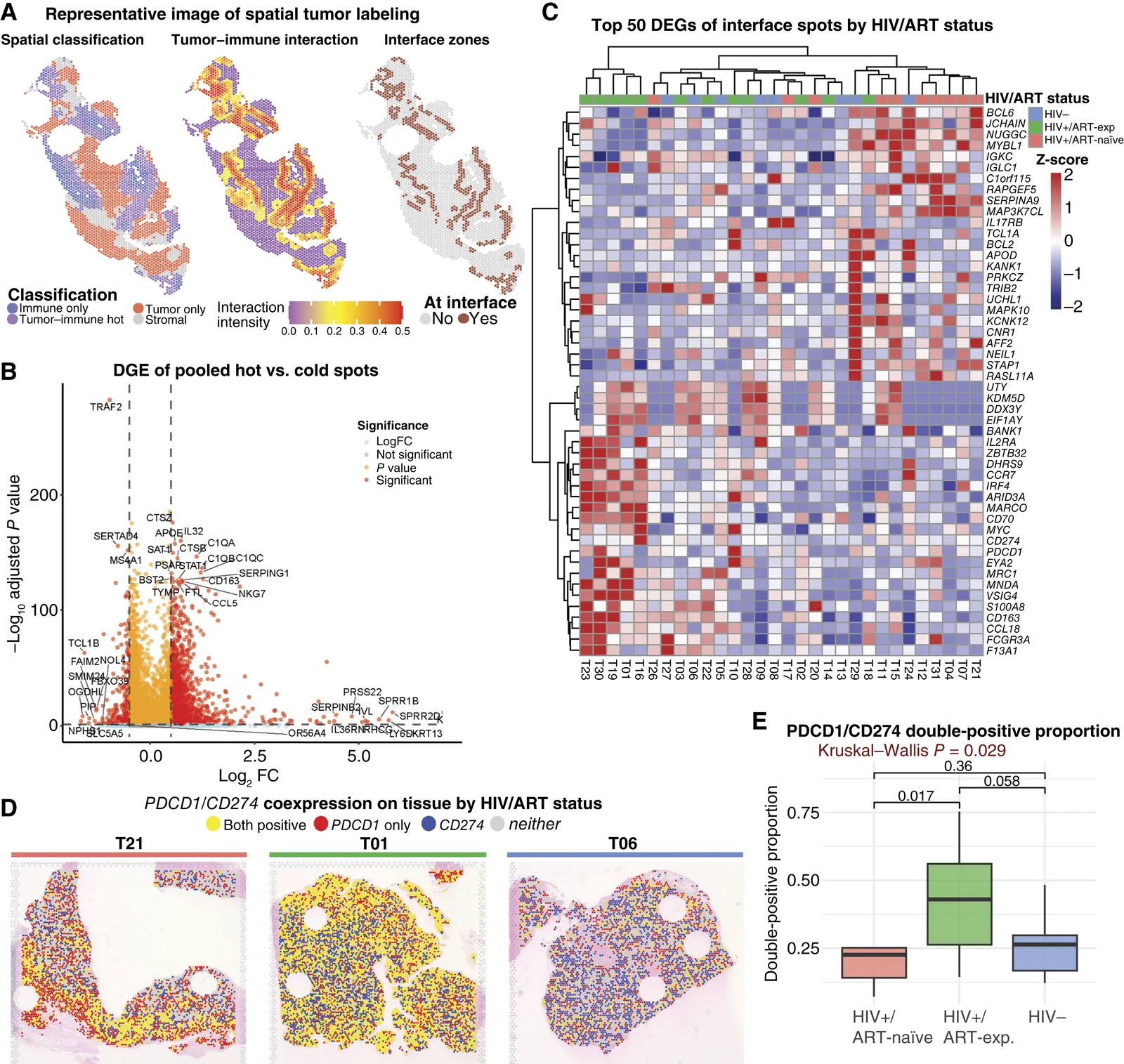
Tumor–immune interface transcriptional programs and PDCD1/CD274 coexpression by HIV/ART status. **A,** Representative tumor showing spot-labeling identifying immune-hot spots by ST. In panel one, spots are colored by classification as immune-only, tumor–immune hot, tumor-only, and stroma. Panel two shows the tumor–immune interaction intensity, highlighting the tumor–immune–hot regions of the tumor. Panel three shows the interface spots, located at the stromal/immune–tumor interface and containing the spots most akin to the tumor–immune–hot neighborhood identified by IMC. Representative spatially labeled (**B**) volcano plot showing meta-analysis of DEGs between immune-hot and immune-cold spots across all tumors. Dashed lines indicate significance thresholds (adjusted *P* < 0.05 and |log_2_ FC| > 0.5). **C,** Heatmap of top 50 DEGs from interface spots stratified by HIV/ART status. Annotation bar indicates HIV/ART status (blue: HIV−; pink: HIV+/ART-naïve; and green: HIV+/ART-exp). Expression values are row-scaled; clustering was performed using the Ward method with correlation distance. **D,** Representative spatial maps showing *PDCD1* and *CD274* coexpression across tumors from each HIV/ART group. Spots are colored by colocalization status: yellow (*PDCD1*+*CD274*+ double-positive), red (*PDCD1*+ only), blue (*CD274*+ only), and gray (neither). Color bars above each image indicate HIV/ART status (pink: HIV+/ART-naïve; green: HIV+/ART-exp; and blue: HIV−). **E,** Quantification of *PDCD1*/*CD274* colocalization by HIV/ART status. Left: proportion of spots double-positive for *PDCD1* and *CD274*. Right: Jaccard index measuring spatial overlap between *PDCD1*+ and *CD274*+ populations. Boxplots show median and IQR; pairwise comparisons by the Wilcoxon rank-sum test with Kruskal–Wallis *P* values shown in red.

To further investigate these immune-driven differences and extend our analysis across the full cohort, including HIV− tumors, we leveraged the transcriptomic readout and broader tissue coverage of ST to redefine immune-hot and immune-cold regions directly from transcriptomic data. Spots were first labeled as immune-hot or immune-cold (tumor-only) based on prior cell-type assignment, and within-tumor DEG meta-analysis comparing immune-hot with immune-cold spots identified 2,032 significant DEGs (adjusted *P* < 0.05 and |log_2_FC| > 0.5; Fig. 2A and B). Hot regions showed upregulation of complement components (*C1QA*, *C1QB*, and *C1QC*), cytotoxic marker *NKG7*, *CCL5*, *CD163*, *STAT1*, and *APOE*, and cathepsins (*CTSB* and *CTSL*). *TRAF2*, a key NF-κB signaling mediator that promotes M2 macrophage polarization and immunosuppression, was the most significantly upregulated gene in cold/tumor-only regions, alongside *SERTAD4*, *TCL1B*, and *FAIM2*.

Extending this transcriptomically resolved classification to the tumor level and incorporating spatial organization, each tumor was assigned a *z*-score–normalized composite spatial score integrating immune infiltration, tumor–immune mixing, immune dispersion, and interface thickness, and tumors were labeled as spatially hot (scoring above 0) or spatially cold (scoring below 0; Fig. 2A; Supplementary Fig. S8A). Although not statistically significant, HIV+/ART-exp tumors were more frequently hot (75%) compared with HIV− (33%) and HIV+/ART-naïve (40%; Supplementary Fig. S8B). Hierarchical clustering of top 50 whole-tumor DEGs by hot versus cold tumor comparison identified two spatially hot clusters with opposing gene expression, as well as several spatially cold tumors sharing expression with hot tumors, indicating heterogeneity in immune architecture not fully captured by HIV/ART status or spatial labeling (Supplementary Fig. S8C). Hot tumors, particularly four HIV+/ART-exp cases, showed upregulation of macrophage markers (*CD68*, *CD33*, and *MARCO*), inhibitory receptor *LAIR1*, lysosomal components (*CTSZ*, *ATP6V1B2*, *ATP6V0C*, *GAA*, and *GNS*), and T-cell genes (*CD4*, *PDCD1LG2*, *GZMA*, and *GZMK*). Cold tumors showed elevated *IL6*, epithelial markers (*KRT17* and *SLPI*), developmental/stem-like genes (*MIXL1*, *VANGL2*, and *EPHB1*), and antiapoptotic *BCL2L10*. The incomplete separation between hot and cold tumors at the whole-tumor level suggests that intratumoral heterogeneity dilutes immune-driven transcriptomic signals when bulk tumor expression is considered.

### ART-experienced tumors harbor immunosuppressive activity at the tumor–immune interface

To overcome this dilution and isolate the regions most representative of active tumor–immune engagement, we focused on spots located at the tumor–stroma interface, in which the IMC-derived tumor–immune–hot neighborhood was localized, and differential gene expression was performed by HIV/ART status (Fig. 2C). Hierarchical clustering revealed partial separation by HIV status, with two distinct clusters of HIV+/ART-exp cases and one cluster of HIV+/ART-naïve cases. HIV− tumors clustered with both HIV+ groups and formed a small cluster. HIV+/ART-exp interface spots showed elevated myeloid genes (*MRC1*, *FCGR3A*, *S100A8*, *MNDA*, and *F13A1*) and immunosuppressive M2/TAM markers (*CD163*, *MARCO*, and *DHRS9*), immune checkpoints (*CD274* and *PDCD1*), *CCL18*, *IL2RA*, and *CD70*. HIV+/ART-naïve interface spots exhibited B-cell gene enrichment including *BCL6*, immunoglobulin components (*IGLC1*, *IGKC*, and *JCHAIN*), *SERPINA9*, *TCL1A*, *MYBL1*, *MYC*, and *IRF4*. HIV− tumors showed a heterogeneous pattern distributing across clusters.

### PDCD1/CD274 coexpression is significantly enriched in ART-exp tumors

Given the enrichment of targetable immunosuppressive markers *PDCD1* and *CD274* at the immune interface of HIV+/ART-exp tumors, spatial coexpression was investigated (Fig. 2D). HIV+/ART-exp tumors showed abundant double-positive spots indicating extensive *PDCD1*/*CD274* coexpression. HIV+/ART-naïve tumors displayed predominantly single-positive populations with limited coexpression. HIV− tumors showed an intermediate pattern with dispersed single-positive populations and modest coexpression. HIV+/ART-exp tumors had significantly higher double-positive proportions compared with HIV+/ART-naïve (*P* = 0.017) and trended higher than HIV− (*P* = 0.058; Kruskal–Wallis *P* = 0.029, Fig. 2E).

To validate these transcriptomic findings at the protein level, we examined PD-1 and PD-L1 coexpression in the previously published IMC cohort. At single-cell resolution, PD-1 and PD-L1 were only weakly correlated (Spearman ρ = 0.48), consistent with expression on largely distinct cells. Aggregating IMC cells into 55-μm pseudo-spots to approximate Visium resolution increased this correlation to ρ = 0.89, indicating that PD-1/PD-L1 coexpression is organized at the neighborhood scale rather than at single-cell contact (Supplementary Fig. S9). However, neither the proportion of double-positive pseudo-spots nor any single-cell colocalization or cell–cell distance metric differed by HIV/ART status. Because IMC was performed on tissue microarray cores spanning approximately 1 mm^2^ each (≈100 Visium spots), the tissue area sampled per tumor was roughly two orders of magnitude smaller than the 11 × 11 mm Visium capture area (∼14,000 spots). These data confirm that PD-1/PD-L1 coexpression occurs within shared neighborhoods at the protein level but indicate that IMC sampling was insufficient to resolve the between-group difference in coexpression frequency detected by ST.

### Tumor-dominant spots show transcriptomic overlap across HIV/ART groups with divergence driven by ART exposure

**Figure 3.**
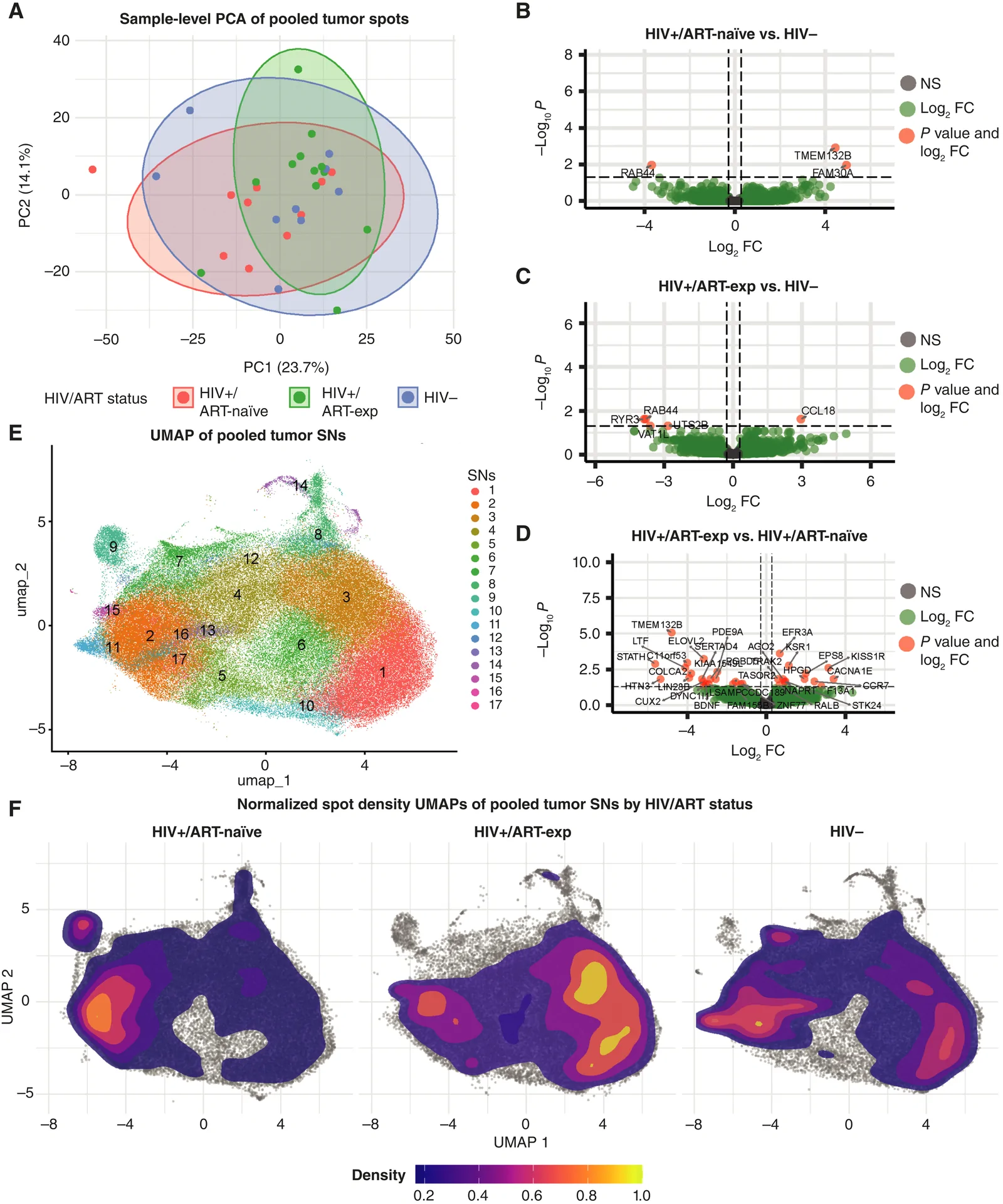
Tumor-intrinsic transcriptomic profiles and SN identification across HIV/ART groups. **A,** PCA of tumor-only spots aggregated at the sample level, with each point representing one tumor. Samples are colored by HIV/ART status (HIV+/ART-naïve in pink, HIV+/ART-exp in green, and HIV− in blue) with 95% confidence ellipses. PC1 and PC2 account for 23.7% and 14.1% of variance, respectively. **B–D,** Volcano plots of pseudobulk differential gene expression between (**B**) HIV+/ART-naïve vs. HIV− tumors, (**C**) HIV+/ART-exp vs. HIV− tumors, and (**D**) HIV+/ART-exp vs. HIV+/ART-naïve tumors. Significant genes (*P*.adj < 0.05 and |log_2_ FC| > 0.28) in red and labeled; genes meeting only the FC threshold are in green. Dashed lines indicate significance cutoffs. **E,** UMAP of pooled tumor-only spots from full cohort, colored by shared SN identity (clusters 0–18) identified across all tumors. **F,** Normalized two-dimensional kernel density estimate of tumor-only spots split by HIV/ART status, indicating the weighted distribution of SN presence as seen in **F**. Color intensity represents local dot density on the UMAP. NS, nonsignificant.

Having characterized HIV/ART-associated differences in the spatial immune architecture and tumor–immune interface, we next investigated tumor-intrinsic biology to determine whether the lymphoma cells themselves harbor distinct programs across HIV/ART contexts. To isolate tumor-intrinsic features from microenvironmental contributions, spots labeled as tumor-dominant were selected for downstream analysis (*n* = 31 tumors, one tumor excluded because of histiocyte-rich histology obscuring isolation of tumor-dominant spots). Sample-level PCA of tumor-dominant spots showed substantial overlap among HIV/ART groups, with largely overlapping 95% confidence ellipses (Fig. 3A). Pairwise pseudobulk differential expression identified relatively few significant genes across all three comparisons, consistent with this transcriptomic overlap (Fig. 3B–D). The greatest number of DEGs was observed between HIV+/ART-exp and HIV+/ART-naïve tumors, with significant genes implicating lymphocyte trafficking (*CCR7*), RAS/MAPK signaling (*KSR1*), and immune regulatory pathways (*HPGD*; Fig. 3D). *RAB44*, a GTPase implicated in immune cell differentiation, was the only gene significantly differentially expressed in both HIV+ versus HIV− comparisons.

Although bulk tumor-dominant transcriptomes did not reflect comprehensive molecular subtyping, this overall similarity suggested that broad differences in tumor biology were unlikely to dominate downstream comparisons. To address the most established axis of DLBCL molecular heterogeneity, COO, we performed transcriptomic COO classification using gneSeqCOO and compared it with IHC-based Hans algorithm assignment. Each tumor-dominant spot was scored individually with gneSeqCOO, and tumors were assigned to the majority spot-level call (Supplementary Fig. S10A). Overall concordance was 68.8% (75% for GC and 58.3% for non-GC; Supplementary Fig. S10B). Predicted COO distribution did not differ significantly across HIV/ART groups (Supplementary Fig. S10C). Among TIME cell-type proportions, only CD8^+^ T cells differed significantly by COO, with unclassified tumors showing modestly fewer CD8^+^ T-cell spots than activated B cell (ABC) and germinal center B cell (GCB) tumors, although median proportions were <0.05% across all three groups (Supplementary Fig. S10D). COO was additionally not associated with sample-level PCA structure of tumor-dominant spots or with spatial immune classification (Supplementary Fig. S10E and S10F).

### Unsupervised clustering identifies 17 shared SNs with group-specific enrichment

Having established that bulk tumor-dominant transcriptomes are broadly comparable across HIV/ART groups, we next investigated whether tumor-intrinsic differences emerge at the level of subclonal architecture. To identify shared tumor-intrinsic features across HIV/ART groups, we performed unsupervised clustering on pooled tumor-only spots, identifying 17 transcriptionally distinct shared SNs (Fig. 3E). Density mapping revealed differential representation across groups: HIV+/ART-naïve and HIV+/ART-exp tumors showed concentrated density in opposing UMAP regions, whereas HIV− tumors displayed a more diffuse distribution (Fig. 3F). Several niches were dominated by a single group: SNs 11 and 13 were almost exclusively HIV−, SNs 15 to 17 were predominantly HIV+/ART-exp, and SNs 9 and 12 were HIV+/ART-naïve, whereas SN14 was notably shared between HIV− and HIV+/ART-exp tumors (Supplementary Fig. S11A). Other SNs contained spots from all three groups in balanced proportions (Supplementary Fig. S11A).

To characterize the top distinct oncogenic programs active within each SN, enrichment analysis was performed using the MSigDB C6 oncogenic gene sets on the DEGs per SN (Supplementary Fig. S11B). The top enriched signature varied across niches, with several shared pathways observed. Ribosomal protein–related signatures (RPS) were the top-ranked pathway in multiple niches (1, 2, 3, 6, 9, and 15). In the HIV− dominated SNs (11 and 13), the top ranked pathways were *MTOR* and *EIF4E* signatures, respectively. In the HIV+/ART-exp enriched SNs (15–17), the top pathways included the shared RPS signature, a chronic wound healing response signature (CSR_Late), and a *MYC*-associated signature, respectively. Lastly, in the HIV+/ART-naïve dominated SNs (9, 12), the top ranked pathways were again the RPS signature and a *LTE2* signature. Interestingly, SN14, shared by HIV− and HIV+/ART-exp tumors, was enriched for an embryonic stem cell signature (ESC). Other identified pathways in shared SNs include STK33_NOMO_UP, a retinoblastoma gene signature (RB_P107_DN.V1_UP), and a *KRAS* signature (KRAS.KIDNEY_UP.V1_DN).

Comparison of oncogenic signature enrichment between SNs enriched in HIV+ and HIV− tumors revealed that HIV+ tumors were preferentially enriched for *STK33*, *RPS14*, *NFE2L2*, *MEK*/*ERK*, *EGFR*, *ERBB2*, and *E2F1* signatures, whereas HIV− SNs were preferentially enriched for *EIF4E*, *PTEN*, *IL2*, *JAK2*, *MTOR*, and *TBK1*-associated signatures (Supplementary Fig. S11C). Hierarchical clustering of pathway enrichment across HIV/ART groups further revealed distinct patterns of intragroup heterogeneity. HIV+/ART-exp SNs showed the broadest enrichment and the greatest pathway overlap across niches, indicating relative similarity among these tumors. HIV+/ART-naïve SNs displayed comparably broad enrichment overall, but with many signatures appearing in only one or two niches, pointing to greater dissimilarity across tumors. HIV− SNs were enriched across markedly fewer pathways and fewer niches, reflecting the greatest heterogeneity of the three groups (Supplementary Fig. S11D).

### Per-tumor SN analysis reveals divergent oncogenic programs and polycomb complex preferences across HIV/ART groups

**Figure 4.**
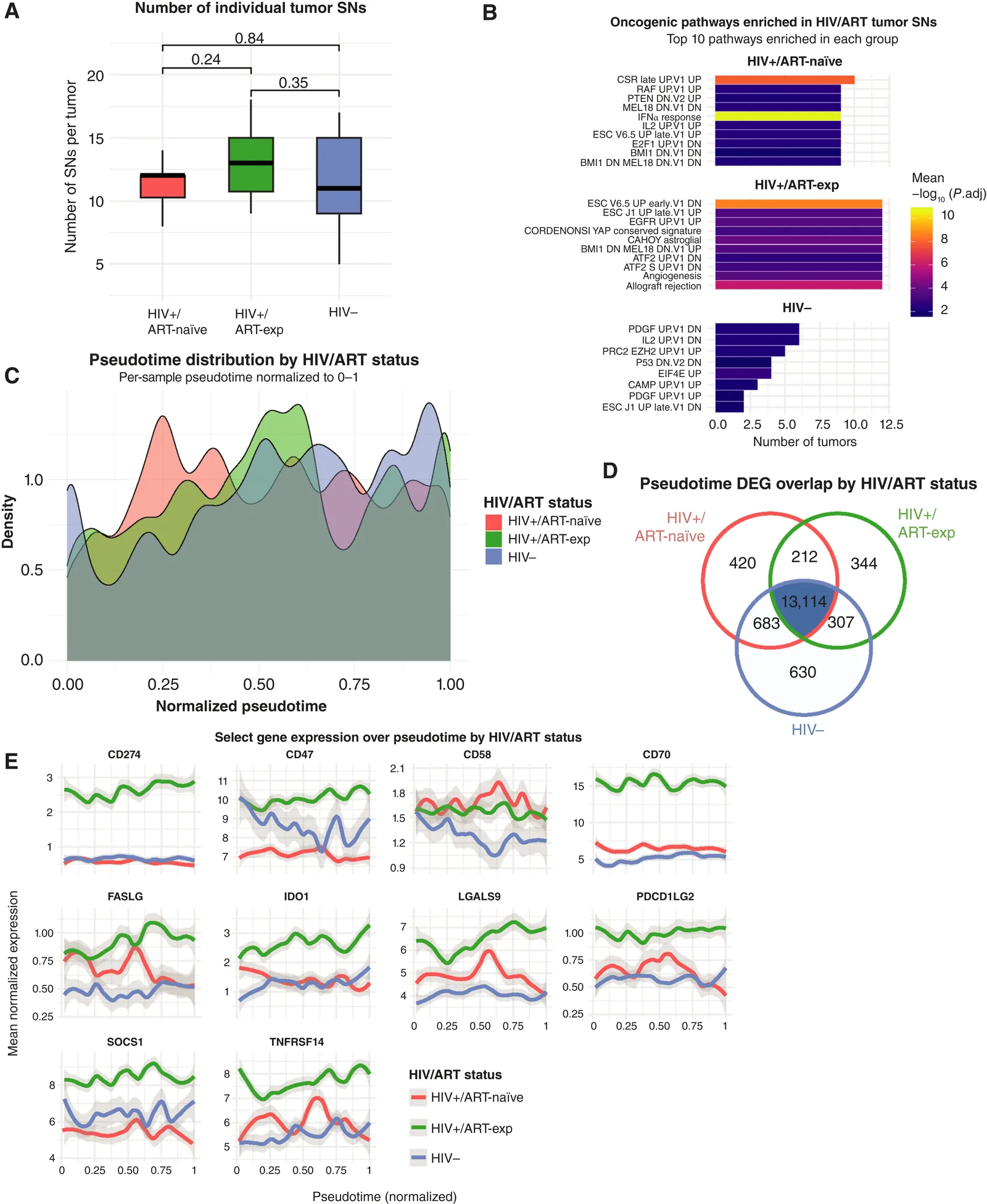
Pseudotime developmental trajectories and immune evasion gene dynamics by HIV/ART status. **A,** Box and whisker plot of the number of tumor SNs identified per tumor, grouped by HIV/ART status. Wilcoxon rank-sum pairwise *P* values are shown above brackets. **B,** Top 10 enriched MSigDB C6 oncogenic signatures across tumor subclones within each HIV/ART group. Bar length indicates the number of tumors in which each pathway was enriched, and color represents the mean log_10_ adjusted *P* value across those tumors. **C,** Density plot of per-sample normalized pseudotime distributions by HIV/ART status, with overlapping densities shown as shaded regions. **D,** Venn diagram of genes significantly associated with pseudotime (Spearman correlation, q < 0.05 in ≥1 tumor per group), showing the number of unique and shared pseudotime-associated genes across HIV/ART groups. **E,** LOESS-smoothed line plots of mean normalized expression over normalized pseudotime for select genes of interest by HIV/ART status. Shaded ribbons represent CIs of the LOESS fit.

Although shared-niche clustering captured cohort-wide structure, pooling across tumors risks obscuring tumor-specific subclonal architecture by averaging over biologically heterogeneous samples. To better resolve intratumoral heterogeneity, we next identified SNs within each individual tumor and aggregated pathway enrichments by HIV/ART status. The number of SNs per tumor was comparable across groups (median 11–13; Fig. 4A), indicating similar overall levels of intratumoral transcriptional heterogeneity regardless of HIV/ART status. However, the oncogenic programs enriched within these niches diverged between groups. SNs identified in HIV+/ART-naïve tumors were enriched for IFNα response, *IL2* signaling, *E2F1*-driven cell-cycle progression, and PRC1 dysregulation (*BMI1*/*MEL18*). HIV+/ART-exp SNs were characterized by PRC1 dysregulation, stem cell–associated programs, *ATF2* stress response signaling, and angiogenesis and allograft rejection signatures. HIV− SNs were preferentially enriched for PRC2/*EZH2*-related chromatin remodeling, *PDGF* signaling, *TP53* pathway downregulation, and *EIF4E* translational control (Fig. 4B).

### Pseudotime analysis reveals HIV/ART-dependent developmental trajectories

The enrichment of stem cell and polycomb repressor programs in HIV+ tumors suggested that HIV/ART status may influence not only oncogenic pathway activity but also tumor differentiation state. To investigate this, we performed pseudotime analysis to order tumor spots along inferred developmental trajectories. Although trajectory complexity metrics (branch points, leaves, and partitions) showed no statistically significant differences across groups (Supplementary Fig. S12), normalized pseudotime density distributions differed by HIV/ART status (Fig. 4C). HIV+/ART-naïve tumors showed prominent peaks in early pseudotime, HIV+/ART-exp tumors displayed a dominant peak near the midpoint with secondary peaks at early and late positions, and HIV− tumors exhibited prominent peaks at mid and late pseudotime with increasing density. This ordering indicates that HIV− tumors exhibit transcriptomic profiles associated with more differentiated states, whereas HIV+/ART-naïve tumors are less differentiated, and HIV+/ART-exp tumors occupy intermediate positions. Because these trajectories were compared between HIV/ART groups rather than between molecular subtypes, COO is unlikely to account for the observed differences; the cohort was predominantly GCB (20 of 31 tumors, with four assigned ABC), and predicted COO did not differ significantly across HIV/ART groups (Supplementary Fig. S10C). The trajectories therefore largely describe transcriptional variation arising within GCB tumors and are interpreted as an inferred transcriptional gradient of tumor differentiation and immune engagement state rather than a genetically defined developmental sequence.

To identify the transcriptional programs driving these trajectories, we next examined which genes varied significantly with pseudotime in each group. The majority of genes significantly associated with pseudotime were shared across all three HIV/ART groups (13,114 genes), with HIV− tumors having the largest unique set (630), followed by HIV+/ART-naïve (420) and HIV+/ART-exp (344; Fig. 4D).

### Immune evasion gene dynamics diverge across HIV/ART groups over pseudotime

Given that HIV and ART are known to reshape both systemic and local immune landscapes, and that immune checkpoint and antigen presentation pathways are increasingly therapeutically actionable in DLBCL, we next focused on how immune-related genes evolve along these developmental trajectories. The expression of select immune checkpoint, antigen presentation, and immune regulatory genes over normalized pseudotime revealed striking divergence across groups (Fig. 4E). Nearly all checkpoint genes had higher expression in HIV+/ART-exp tumors across pseudotime, including *CD274* (PD-L1), *PDCD1LG2* (PD-L2), *CD70*, *LGALS9*, and *TNFRSF14*, for which expression increased across pseudotime, whereas expression in HIV+/ART-naïve tumors peaked at mid-pseudotime and gradually decreased.

*CD47*, the macrophage “don’t eat me” signal, was highly divergent: expression increased over pseudotime in HIV+/ART-exp tumors but decreased in HIV− tumors. *CD58* showed a distinct loss in expression over pseudotime in HIV− tumors, with greatest expression in HIV+/ART-naïve tumors. *IDO1* was highest in HIV+/ART-exp tumors and increased over pseudotime in both HIV+/ART-exp and HIV− tumors. The proapoptotic ligand *FASLG* showed a marked decrease in expression over pseudotime in HIV+/ART-naïve tumors, whereas expression increased in HIV+/ART-exp tumors and remained stably low in HIV− tumors.

These patterns support the notion that HIV+/ART-naïve and HIV+/ART-exp tumors are not subsets of a shared HIV+ transcriptomic program but harbor distinct microenvironmental pressures and evolutionary trajectories. HIV+/ART-exp tumors seem to evolve toward increasing checkpoint ligand expression and macrophage-focused evasion, whereas HIV− tumors develop T cell–specific evasion strategies, including *CD58* loss and PRC2-mediated MHC class II silencing.

### TMB-high and TMB-low tumors harbor distinct oncogenic programs

Because TMB has emerged as a determinant of immune engagement and therapeutic response in DLBCL, and because HIV+/ART-exp DLBCL in our cohort has been previously reported to harbor elevated TMB (24), we next asked whether TMB stratification reveals tumor-intrinsic patterns distinct from those defined by HIV/ART status. This exploratory analysis was performed on the subset of six tumors with paired exome sequencing. With only three tumors per group, these comparisons are underpowered and are presented as exploratory and hypothesis-generating rather than definitive. Pseudobulk differential expression between TMB-high and TMB-low tumors identified a modest number of significant genes (Supplementary Fig. S13A). GSEA revealed divergent pathway programs: TMB-high tumors were enriched for MYC activation, ribosomal stress, innate immune signaling, and polycomb dysregulation, whereas TMB-low tumors showed dependence on multiple KRAS-related pathways and p53 pathway downregulation (Supplementary Fig. S13B).

### Divergent evolutionary trajectories by TMB status

TMB-high tumors resolved into nine SNs with a pseudotime trajectory traversing multiple niches (Supplementary Fig. S13C). TMB-low tumors contained 12 SNs with more complex trajectory architecture, although with a greater number of sequenced tumor-dominant spots (5,141 vs. 1,764; Supplementary Fig. S13D).

In TMB-high tumors, the top pseudotime-associated genes revealed a dominant pattern of high early expression of proliferative and replication machinery (MCM helicase complex members, cell-cycle regulators, protein-folding chaperones, and GC B-cell transcription factors) with progressive decline, giving way to increasing expression of chemokines and their receptors (*CXCR4*, *CCL5*, and *CCL19*), T-cell markers (*GZMK* and *TRBC1*), and antigen presentation machinery (*B2M*; Supplementary Fig. S14). This pattern suggests a transition from proliferative to immune-engaged states, potentially driven by increasing neoantigen diversity.

TMB-low tumors displayed a contrasting pattern (Supplementary Fig. S15). A small cluster of genes with high early expression that declined included histone and chromatin remodeling genes (*HIST1H1C*, *HIST1H1B*, and *HMGB2*) and the GC marker *LRMP*. The dominant pattern was progressive accumulation of extracellular matrix, stromal, and myeloid-associated genes, including collagens (*COL1A2*, *COL3A1*, *COL5A1*, *COL5A2*, and *COL6A3*), matrix components and remodeling enzymes (*FN1*, *LUM*, *DCN*, *THBS2*, and *ADAMTS2*), complement components (*C1S*, *C1R*, and *SERPING1*), and myeloid/macrophage markers (*CD14*, *LYZ*, and *SPP1*). Rather than engaging adaptive immunity, TMB-low tumors seem to evolve toward a fibrotic, myeloid-enriched microenvironment.

Analysis of individual genes reinforced these divergent trajectories (Supplementary Fig. S13E). *CD47* was higher in TMB-high tumors but decreased over pseudotime, while increasing in TMB-low tumors, suggesting that as neoantigen-driven immune pressure intensifies, TMB-high tumors may lose reliance on innate immune evasion whereas TMB-low tumors increasingly depend on it. *CD70* was similarly higher in TMB-high tumors.

## Discussion

In this study, we integrated IMC, ST, and whole-exome sequencing to characterize the spatial immune architecture and tumor-intrinsic biology of DLBCL across a spectrum of HIV-associated immune dysregulation. Our findings suggest that each HIV/ART context is associated with a distinct DLBCL immune architecture and subclonal program that may be shaped by the degree of immune reconstitution. The immunosuppressive, checkpoint-enriched microenvironment of HIV+/ART-experienced tumors, the non–tumor-targeting T-cell responses in HIV+/ART-naïve tumors, and the T cell–specific evasion strategies in HIV− tumors each carry therapeutic implications.

Building on our prior work demonstrating HIV/ART-associated alterations in DLBCL TIME cellular composition and T-cell exhaustion (6), we identified a tumor–immune–hot neighborhood by IMC that associated with inferior survival in HIV+ DLBCL. Absolute immune cell proportions reported here differ from those in our earlier IMC study (6) because the two analyses included partially overlapping but nonidentical patient subsets and used distinct platforms (ST vs. IMC); as such, these datasets should be viewed as complementary rather than directly comparable. This finding contrasts with HIV− DLBCL cohorts in which immune infiltration has been associated with favorable prognosis (44), suggesting that immune infiltration may have fundamentally different functional consequences in the setting of HIV-associated immune dysregulation. Recent spatial profiling studies in HIV− DLBCL have also identified functionally significant cellular neighborhoods, including macrophage-enriched TIME categories characterized by CD163^+^ macrophages, elevated IDO-1 and LAG-3, and immune evasion signatures (34, 35). Our tumor–immune “hot” neighborhoods share key transcriptional features with these macrophage-enriched niches.

ST extended these observations by demonstrating that, despite the heterogeneity observed in the HIV+/ART-exp group, these tumors harbor significantly greater macrophage infiltration, and a subset further exhibits an immunosuppressive microenvironment at the tumor–immune interface, characterized by M2/TAM markers, immune checkpoint expression, and significantly increased *PDCD1*/*CD274* coexpression. Thus, ART-mediated immune reconstitution may promote an actively immunosuppressive DLBCL environment, potentially through increased *TRAF* activity (45) or *SUNCR1*-mediated M2/TAM polarization (46–48). Critically, enrichment of immunosuppressive macrophages has also been identified in a single-cell study of HIV+ DLBCL from a Chinese population, indicating that this tumor biology likely reflects HIV-induced immunosuppression and is not unique to our cohort demographics (33). In contrast, HIV+/ART-naïve interface spots showed GC B-cell signatures (*BCL6*, *SERPINA9*, and immunoglobulins), consistent with the observation that these tumors, arising under unrestrained immunosuppression, do not evolve actively immunosuppressive or evasive microenvironments.

Tumor-intrinsic analyses revealed that although bulk transcriptomic profiles overlapped substantially across HIV/ART groups, unsupervised clustering identified SNs with group-specific enrichment, including divergent involvement of polycomb repressor complexes: PRC1/*BMI1* enrichment in both HIV+ groups and PRC2/*EZH2* enrichment in HIV− tumors. *BMI1* plays a critical role in maintaining HIV latency (49), and overexpression is associated with unfavorable prognosis in DLBCL through repression of differentiation and cell cycle checkpoint programs (50, 51). These observations suggest that PRC1/*BMI1* overexpression in HIV+ DLBCL may be a consequence of HIV-driven epigenetic reprogramming co-opted during lymphomagenesis, although further work is needed. PRC2/*EZH2* enrichment in HIV− tumors is consistent with *EZH2*-mediated silencing of MHC class II as a canonical immune evasion mechanism in GCB lymphomagenesis, potentially arising as a result of T cell–mediated selective pressure in immunocompetent settings (52).

Pseudotime analysis further supported these immune-driven distinctions. The ordering of tumors from early pseudotime (HIV+/ART-naïve) through intermediate (HIV+/ART-exp) to late (HIV−) positions suggests that intact immune surveillance promotes clonal selection and differentiation, whereas immunosuppression permits persistence of less differentiated tumor populations. Critically, HIV+/ART-exp tumors showed the highest and frequently increasing expression of checkpoint ligands, including *CD274*, *PDCD1LG2*, *CD70*, *LGALS9*, and *IDO1*, over pseudotime. HIV has been shown to induce PD-L1 expression on B cells via virion-associated CD40L/CD40 interaction, and CD19^+^PD-L1+ B cells are significantly elevated years before HIV/acquired immunodeficiency syndrome–associated NHL diagnosis (53), suggesting that checkpoint ligand upregulation may represent an early and sustained feature of HIV-associated B-cell transformation. The divergent dynamics of *CD47*, increasing in HIV+/ART-exp but decreasing in HIV− tumors, and *CD58*, lost over pseudotime exclusively in HIV− tumors, further underscore that these tumors employ fundamentally different survival strategies dictated by the immune pressures they face.

Immune architectures and evasion strategies carry direct therapeutic implications. The immunosuppressive, checkpoint-enriched microenvironment identified in a substantial subset of HIV+/ART-exp tumors, characterized by extensive *PDCD1*/*CD274* coexpression and enrichment for high TMB, provides multilayered biological rationale for immune checkpoint blockade in PWH, a population historically excluded from such trials (10, 11), although recent data suggest these therapies are safe and effective (54, 55). The prominence of immunosuppressive macrophages and increasing *CD47* expression further supports exploration of macrophage-targeting strategies, including CD47–SIRPα inhibitors and CSF1R antagonists, which are currently advancing in clinical development for DLBCL and Hodgkin lymphoma (56–58). In HIV− tumors, the marked loss of *CD58* over pseudotime is consistent with recent findings identifying *CD58* loss in relapsed/refractory HIV− DLBCL subclones, which confers resistance to chimeric antigen receptor T (CAR-T) therapies (23, 59). Importantly, this was identified exclusively in HIV− tumors in our cohort, indicating that CD58-targeting strategies may not be as relevant in HIV+ DLBCL.

Exploratory TMB analysis, although limited by sample size, reinforced these patterns. TMB-high tumors, which in our cohort are more frequently HIV+/ART-exp (24), evolved toward immune-engaged states with increasing expression of chemokines, T-cell markers, and antigen presentation machinery, whereas TMB-low tumors developed fibrotic, myeloid-enriched microenvironments. The enrichment of KRAS-related pathways in TMB-low tumors aligns with recent identification of RAS–MAPK pathway deregulation in B-cell lymphomas and may have implications for applying RAS–MAPK signaling inhibitors (60). These findings suggest that TMB and HIV/ART status interact to shape evolutionary trajectories and that combined consideration of both variables may be necessary for optimal therapeutic selection, although TMB alone is insufficient to predict checkpoint response without considering immune microenvironmental and spatial architecture (61, 62).

Despite the advances made in this study, several limitations remain. Visium’s 55 μm spot size lacks single-cell resolution, but integration with paired single-cell IMC in 26 cases enabled cross-platform validation across modalities with distinct tissue sampling and molecular output. As a further consequence of this resolution limit, malignant B cells could not be distinguished from nonmalignant B cells within tumor-dominant spots; background B cells that may participate in tumor–host interactions are therefore included in these analyses and may influence downstream tumor-intrinsic, SN, and pseudotime results. Our cohort is drawn from a single site in Malawi, which, although limiting generalizability, provides an essential window into HIV-associated DLBCL in an endemic setting. Most critically, DLBCL is a highly heterogeneous disease, and we were unable to fully account for features such as mutational landscape and genetic subtyping; yet, despite these variabilities, we identify reproducible patterns by HIV/ART status alone, supporting its utility as a clinically relevant and accessible stratifier of underlying tumor immune biology. Finally, response to ART is heterogeneous, and the distinction between immunologic responders and nonresponders, only partially captured by the duration-based cutoff used here, represents an important axis of variation that will require larger, longitudinally characterized cohorts to resolve. Given the modest size of each subgroup, the group-associated patterns described here should be interpreted as features observed within these subgroups and as hypotheses for future investigation rather than as defining or exclusive characteristics of each HIV/ART group. Future work in larger, multisite cohorts incorporating single-cell spatial platforms, comprehensive molecular subtyping, and longitudinal HIV clinical data will be needed to validate these findings across diverse populations. Defining the clinical and virologic correlates that identify the immunosuppressive, checkpoint-enriched HIV+/ART-experienced subpopulation will be particularly important for patient selection in future immunotherapy trials.

In conclusion, this study provides the first integrated multiplatform spatial characterization of DLBCL across a spectrum of HIV-associated immune dysregulation. Our findings suggest that systemic immune status broadly influences the selective pressures that shape lymphoma development, including the TIME and the evolution of immune evasion strategies. Together, the results support further investigation of immune checkpoint blockade and macrophage-targeting approaches in HIV+/ART-exp DLBCL and underscore the urgent need to include PWH in molecular studies and immunotherapy trial design.

## Supplementary Material

Supplemental File 1 IMC Antibody Panel per reviewer request from previous publication (ref 6)

Supplemental Figure S1 Supplemental Figure S1. Quality control metrics of Visium spatial transcriptomic data.

Supplemental Figure S2 Supplemental Figure S2. Peripheral and intratumoral CD4 T-cell content in relation to immune architecture and spatial classification.

Supplemental Figure S3 Supplemental Figure S3. Peripheral CD4 count and HIV RNA by ART exposure.

Supplemental Figure S4 Supplemental Figure S4. A) Cellular composition of IMC-derived cell type neighborhoods. Stacked bar chart shows the proportional cellular compositions of the six identified cell type neighborhoods. B) Violin plot of the proportional differences of the six cell-type neighborhoods by HIV/ART status. Each dot represents the proportion of the cell-type neighborhood in one ROI from the IMC tissue microarray. The black bar represents the median. P-values are calculated by Wilcoxon rank-sum tests.

Supplemental Figure S5 Supplemental Figure S5. Heatmap of differentially expressed genes by supervised cell type labeling of Visium data for n=32 tumors by pseudobulk.

Supplemental Figure S6 Supplemental Figure S6. Comparison of proportion of tumor immune microenvironment by HIV/ART status.

Supplemental Figure S7 Supplemental Figure S7. Heatmap of differentially expressed genes by PCA-based clusters of HIV+ tumors.

Supplemental Figure S8 Supplemental Figure S8. Spatial classification of immune hot and cold tumors.

Supplemental Figure S9 Supplemental Figure S9. Protein-level validation of PD-1/PD-L1 coexpression by imaging mass cytometry.

Supplemental Figure S10 Supplemental Figure S10. Cell-of-origin classification and its relationship to HIV/ART status, cell-type composition, and spatial immune architecture.

Supplemental Figure S11 Supplemental Figure S11. Oncogenic pathway enrichment across shared subclonal niches stratified by HIV/ART status.

Supplemental Figure S12 Supplemental Figure S12. Pseudotime complexity by HIV/ART status.

Supplemental Figure S13 Supplemental Figure S13. Exploratory analysis of tumor-intrinsic gene expression, subclonal architecture, and pseudotime trajectories by tumor mutational burden.

Supplemental Figure S14 Supplemental Figure S14. Heatmap of the top 50 pseudotime-associated genes in TMB-H tumors.

Supplemental Figure S15 Supplemental Figure S15. Heatmap of the top 50 pseudotime-associated genes in TMB-L tumors.

Supplemental Table S1 Supplemental Table S1. Visium cell type supervised labeling gene markers.

Supplemental Table S2 Supplemental Table S2. Tumor mutational burden (mutations) per tumor.

Supplemental Table S3 Supplemental Table S3. Univariate and multivariate cox hazard regression for HIV+ immune-hot neighborhood presence by both median cut-off and as a continuous variable.

## Data Availability

Visium ST data, including filtered gene by barcode count matrices, cloupe files, image files, and spot alignment files, are available at GSE344506. Exome sequencing data are available at GSE285377. Code is available by request from corresponding author.
