# Supplementary material for: HIV and Antiretroviral Therapy Influence Tumor Microenvironment Architecture, Subclonal Evolution, and Immune Evasion Strategies in Diffuse Large B-cell Lymphoma
Source: Cancer Res Commun. 2026 Oct 22;6(10):2475–91. doi: 10.1158/2767-9764.CRC-26-0343 (PMC13688170; doi:10.1158/2767-9764.CRC-26-0343)
Supplement: Supplementary file 1 [file crc-26-0343_supplemental_table_s1_suppst1.docx]

| **Cell Type** | **Markers** |
| --- | --- |
| B cell (Tumor) | MS4A1, CD79A, CD79B, CD19 |
| CD4+ T cell | CD3E, CD3D, CD3G, CD4 |
| T follicular helper (Tfh) cell | CD3E, CD3G, CD3D, CD4, CXCR3 |
| CD8+ T cell | CD3E, CD3D, CD3G, CD8a |
| Macrophages | MARCO, MSR1, CD68 |
| Endothelial | MCAM, CDH5, VWF |

**Supplemental Table S1. Visium cell type supervised labelling gene markers.**
