# Supplementary material for: HIV and Antiretroviral Therapy Influence Tumor Microenvironment Architecture, Subclonal Evolution, and Immune Evasion Strategies in Diffuse Large B-cell Lymphoma
Source: Cancer Res Commun. 2026 Oct 22;6(10):2475–91. doi: 10.1158/2767-9764.CRC-26-0343 (PMC13688170; doi:10.1158/2767-9764.CRC-26-0343)
Supplement: Supplementary file 2 [file crc-26-0343_supplemental_table_s2_suppst2.docx]

**Supplemental Table 2. Tumor mutational burden (mutations) per tumor.**

| **Tumor ID** | **Mutations** |
| --- | --- |
| T03 | 142 |
| T11 | 141 |
| T14 | 110 |
| T12 | 48 |
| T22 | 41 |
| T29 | 78 |
