# Supplementary material for: HIV and Antiretroviral Therapy Influence Tumor Microenvironment Architecture, Subclonal Evolution, and Immune Evasion Strategies in Diffuse Large B-cell Lymphoma
Source: Cancer Res Commun. 2026 Oct 22;6(10):2475–91. doi: 10.1158/2767-9764.CRC-26-0343 (PMC13688170; doi:10.1158/2767-9764.CRC-26-0343)
Supplement: Supplementary file 3 [file crc-26-0343_supplemental_table_s3_suppst3.docx]

**Supplemental Table 3. Univariate and multivariate cox hazard regression for HIV+ immune-hot neighborhood presence by both median cut-off and as a continuous variable.**

|  | **Model** | **HR** | **95% CI** | **p-value** |
| --- | --- | --- | --- | --- |
| Immune-hot classification (median split; high vs low) | Unadjusted | 2.77 | 1.15–6.69 | 0.023 |
|  | + ART exposure | 3.16 | 1.28–7.80 | 0.013 |
|  | + Rituximab | 2.81 | 1.17–6.78 | 0.021 |
|  | + Age | 2.69 | 1.11–6.51 | 0.029 |
|  | + Cell-of-origin | 3.24 | 1.29–8.13 | 0.012 |
|  | + Age-adjusted IPI | 5.19 | 1.86–14.44 | 0.002 |
| Hot-neighborhood abundance (continuous; HR per doubling) | Unadjusted | 1.44 | 1.01–2.06 | 0.043 |
|  | + ART exposure | 1.58 | 1.09–2.28 | 0.017 |
|  | + Rituximab | 1.46 | 1.03–2.08 | 0.035 |
|  | + Age | 1.46 | 1.01–2.10 | 0.042 |
|  | + Cell-of-origin | 1.62 | 1.11–2.37 | 0.013 |
|  | + Age-adjusted IPI | 2.05 | 1.33–3.16 | 0.001 |
